# Lumping or splitting: seeking the preferred areal unit for health geography studies

**DOI:** 10.1186/1476-072X-4-6

**Published:** 2005-03-23

**Authors:** David I Gregorio, Laurie M DeChello, Holly Samociuk, Martin Kulldorff

**Affiliations:** 1 Department of Community Medicine & Health Care, University of Connecticut School of Medicine, 263 Farmington Ave., Farmington, CT, 06030-6205, USA; 2 Department of Ambulatory Care and Prevention, Harvard Medical School and Harvard Pilgrim Health Care, 133 Brookline Ave, Boston, MA, 02215, USA

## Abstract

**Background:**

Findings are compared on geographic variation of incident and late-stage cancers across Connecticut using different areal units for analysis.

**Results:**

Few differences in results were found for analyses across areal units. Global clustering of incident prostate and breast cancer cases was apparent regardless of the level of geography used. The test for local clustering found approximately the same locales, populations at risk and estimated effects. However, some discrepancies were uncovered.

**Conclusion:**

In the absence of conditions calling for surveillance of small area cancer clusters ('hot spots'), the rationale for accepting the burdens of preparing data at levels of geography finer than the census tract may not be compelling.

## Background

The geographic study of cancer patterns can be an important tool in disease control and prevention [1], as well as a resource for generating hypotheses about pathogenesis [2]. Unfortunately, there is little practical guidance available as to whether or how to select an 'ideal' level of geography for surveillance of events with distinctive spatial autocorrelations [3]. Designating the geo-spatial locations of health events (i.e., 'geocoding') so as to be accurate (within acceptable error), precise (to a desired areal unit of analysis) and 'fit for use' (applicable to other available data) [4] can be vexing, even for those with great skill and experience [4-12]. On the one hand, small areal units containing few at-risk subjects will yield less reliable rates than larger units, whereas on the other hand, large areal units have potential to blur meaningful variation occurring within locales. Communicating and interpreting results that disentangle underlying risks from methodological artifact is important for public health workers and epidemiologists alike.

Procedures for spatial analyses of suspected cancer 'hot spots' [13] may be unnecessary and even inappropriate [14] regarding studies of rate variation across large areas [15], as well as those intended to evaluate resource allocations [16,17]. At the same time, concerns to protect confidentiality of geographically referenced health data by those entrusted to collect and manage surveillance data may effectively eliminate some options for analysis. While the underpinnings of the 'modifiable areal unit problem (MAUP)' have been well described [18,19], there is neither guidance to effectively deal with the problem nor few real examples of whether or how differing aggregation units affect actual results. Armheim's treatment of simulated data suggests fewer disparities across findings with greater aggregation of data [19]. Krieger *et al.*, examining all-cause and selected cause-specific mortality and cancer incidence rates across Massachusetts and Rhode Island, found analyses by block group and census tract performed comparably [20], although tract-level analyses were found to offer greater linkage to area-based socio-economic indicators [21]. Sheehan *et al*. reported few differences for town, zip code or census tract-level analyses of breast cancer incidence across Massachusetts, but noted case counts fluctuated due to various geocoding problems [22].

Here, we address the problem of modifiable areal units while examining breast and prostate cancer incidence during a 5 year interval (1988–92) across Connecticut. Initially, we utilized geographically referenced data furnished us by the CT Tumor Registry to consider differences of incidence and late stage cases according to town and census tract. Evidence of either global or local clustering was evaluated using Oden's Ipop [23] and the spatial scan statistic [24]. Subsequently, we independently ascertaining census block group and exact latitude-longitude coordinates of recorded cases to consider whether greater precision of location modified/enhanced initial findings.

## Results

### Prostate cancer incidence

Table 1 displays summary information for results of the Ipop global clustering test. Examining records aggregated by town, tract, or block group, the Ipop results indicated significant non-random clustering of cases throughout the state. Regardless of the analytic unit considered, approximately 40% of spatial clustering of prostate cancer incidence is attributed to the comparability of occurrences 'among' adjacent geographic locations, with any remaining clustering attributable to the incidence of cases within the given geographic units.

**Table 1:** Ipop global clustering. Case count correlations for the geographic distribution of invasive and late-stage prostate or breast cancer incidence within or among selected areal units of analysis, Connecticut, 1988–92.

Areal Unit	Within %	Among %	p-value
Prostate Cancer Incidence			
Block Group	60.0	40.0	0.0002
Tract	60.1	39.9	0.0002
Town	57.4	42.6	0.0002
Breast Cancer Incidence			
Block Group	72.1	27.9	0.0002
Tract	63.7	36.3	0.0002
Town	75.8	24.2	0.0002
Late Stage Prostate Cancer Incidence			
Block Group	99.7	0.3	0.0008
Tract	87.9	12.1	0.2190
Town	77.0	23.0	0.2740
Late Stage Breast Cancer Incidence			
Block Group	100.7	-0.7	<0.0001
Tract	100.2	-0.2	0.0002
Town	89.1	10.9	0.0846

**Figure 1:**
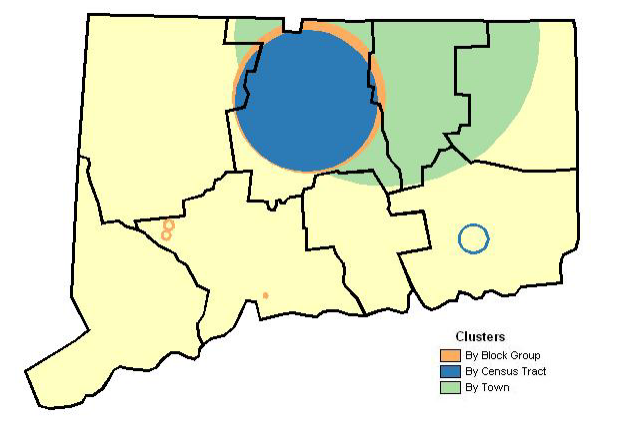
**Prostate cancer incidence. **Geographic variation of prostate cancer incidence according to town, census tract and census block group units, Connecticut 1988–92. Primary clusters are indicated by solid circles and the statistically significant secondary clusters by hollow circles.

Table 2 displays the latitude-longitude coordinates, approximate size, population at risk, numbers of cases and ratio of observed-to-expected cases for locations deemed likely clusters by the spatial scan statistic. Distances between the geographic coordinates of clusters identified for block group-level analyses (reference) and those by town, census tract or individual case coordinates are noted. The spatial scan statistic identified locales throughout the State, Depicted in Figure 1, with potentially significant clustering of prostate cancers. Analysis by block group found four distinct locations with greater than expected incidence, findings for the tract level analysis identified two places and town level results indicated one significant site. The most likely locations for each level of analysis (i.e., primary clusters) depicted as shaded areas are common to North Central Connecticut (centroids of identified areas differed only by 11.1 km) with nearly identical ratios of observed-to-expected cases. The cluster identified at the town level appears more than 4-times the area of those based on census tracts or block groups, although it is much more comparable regarding the respective populations-at-risk (only 20% larger than others) and numbers of cases (11% difference). Additional locations where incidence was determined to be markedly greater than chance (i.e., secondary clusters depicted empty circles), were found in the southwest when analyzed by block group and southeast according to tract-level analysis. There were no significant secondary clusters based on the town-level analysis.

**Table 2:** Spatial scan statistic clusters. Approximate locations with elevated invasive and late-stage prostate or breast cancer incidence according to selected areal units of analysis, Connecticut, 1988–92.

	Geocoded Records	Coordinates (Lat.; Long.)	Area (km^2^)	Population at-risk	Cases in Cluster	0/E	Distance (km)	p-value
Prostate Cancer Incidence								
Block Group	9,028							
1		41.834; -72.727	1,504	254,092	2,651	1.28	Ref.	<0.0001
2		41.311; -72.878	0	345	19	7.62		<0.0001
3		41.472; -73.225	0	148	12	6.97		0.0060
4		41.497; -73.218	0	97	13	5.32		0.0316
Tract	9,825							
1		41.823; -72.735	1,297	238,007	2,673	1.26	1.4	<0.0001
2		41.463; -72.153	0	1,461	33	3.35		<0.0001
Town	10,054							
1		41.995; -72.454	6,104	286,450	2,947	1.22	11.1	<0.0001
Breast Cancer Incidence								
Block Group								
1	11,753	41.182; -73.510	573	85,084	952	1.22	Ref.	11,753
2		41.797; -72.775	115	27,066	391	1.31		0.0048
Tract	10,924							
1		41.137; -73.391	854	147,066	1,554	1.21	11.2	<0.0001
2		41.787; -72.660	0	15	6	162.03		<0.0001
3		41.707; -72.647	87	32,358	401	1.26		0.0228
4		41.795; -72.756	64	20,737	284	1.31		0.0305
5		41.894; -72.368	0	2,137	34	2.34		0.0445
Town	12,518							
1		41.960; -73.311	85	402	24	5.85	88.2	<0.0001
2		41.122; -73.346	584	87,795	1,009	1.14		0.0160
Late Stage Prostate Cancer Incidence								
Place of Residence	7,672							
1		41.486; -73.065	2,057	1,651	549	1.19	2.0	0.0070
2		41.061; -73.458	65	72	41	2.04		0.0135
Block Group	7,672							
1		41.501; -73.078	2,218	1,696	563	1.19	Ref.	0.0029
2		41.054; -73.478	44	61	35	2.05		0.0246
Tract	8,346							
1		41.480; -73.075	1,895	1,596	541	1.22	2.4	<0.0001
Town	8,514							
1		41.489; -73.052	1,959	1,932	644	1.19	2.5	<0.0001
Late Stage Breast Cancer Incidence								
Place of Residence	10,227	No significant clusters detected						
Block Group	10,227	41.666; -72.776	center16	105	center68	1.61	Ref.	0.0092
1								
Tract	10,395	No significant clusters detected						
Town	11,854	No significant clusters detected						

### Breast cancer incidence

Significant global clustering was found at each level of analysis. According to Ipop test results, the percent of incident breast cancer cases clustering among geographic units was somewhat less than that for prostate cancer in results for town (24.2% vs. 42.6%) or block group (27.9 vs. 40.0%), but similar when examined according to census tract (36.3% vs. 39.9%).

**Figure 2:**
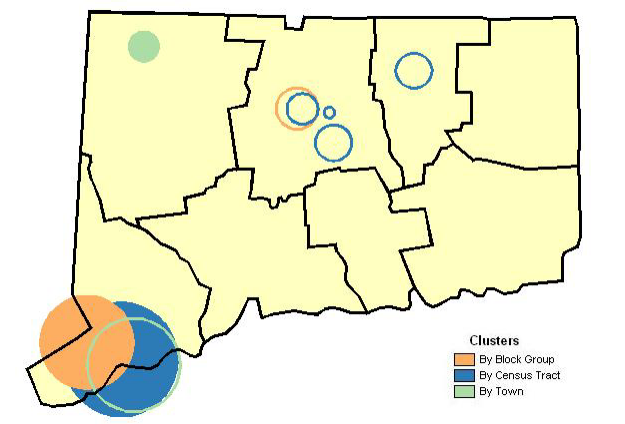
**Breast cancer incidence. **Geographic variation of breast cancer incidence according to town, census tract and census block group units, Connecticut 1988–92. Primary clusters are indicated by solid circles and the statistically significant secondary clusters by hollow circles.

The spatial scan statistic applied to block group level data found two distinct locations, depicted in Figure 2, with greater than expected breast cancer incidence, findings for the tract level analysis found five locations and town level results indicated two locations of possible clustering. There was good agreement regarding proximity and extent of risk between analyses at the block group and tract-level which identified Southwest Connecticut as the most likely location for clusters of incident breast cancers. Analysis by census tract identified a potential cluster with 49% greater area, 63% more cases and 73% larger population at risk than results for analysis by bock group. Those findings, by comparison, differed noticeably from the town-level analysis that identified the primary incidence cluster as single Northwestern Connecticut town (88 km from the center of the most likely cluster identified at the block group level) with a nearly 6-fold ratio of observed-to-expected cases. The town-level analysis yielded a secondary cluster with the locale of primary clusters found by the block group and tract analyses. The latter analyses, in turn, produced significant secondary clusters within North Central Connecticut.

### Proportion of late stage prostate cancer

Results of the Ipop statistic for tract and town level analyses did not reveal global clustering of late-stage prostate cancers, but significant, albeit minimal clustering (i.e., only 0.3% of clustering was attributed to cases adjacent block groups) was indicated in analysis by block group.

**Figure 3:**
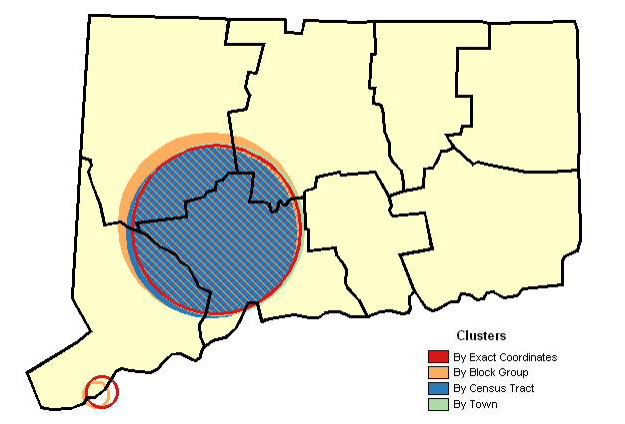
**Late stage prostate cancer incidence. **Geographic variation in proportion of late stage prostate cancer diagnoses according to town, census tract, census block group and exact place of residence units, Connecticut, 1988–92. Primary clusters are indicated by solid or hatch marked circles and the statistically significant secondary clusters by hollow circles.

The spatial scan statistic using data for exact location of residence found two locations with proportions of late-stage cases significantly exceeding the statewide level; cases aggregated by block group revealed two locations while tract and town level analyses each produced one significant location. Results for primary clusters analyzed according to block group, tract, town, and exact place of residence, as illustrated in Figure 3, yielded results with remarkable comparability regarding approximate location, affected areas, populations at risk, case counts and estimated effects.

### Proportion of late stage breast cancer

**Figure 4:**
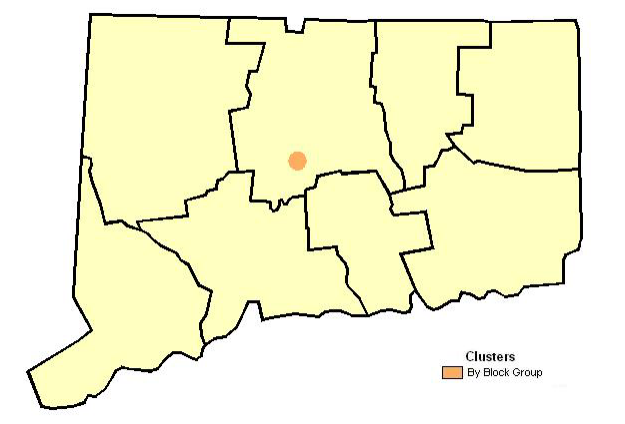
**Late stage breast cancer incidence. **Geographic variation in proportion of late stage breast cancer diagnoses according to town, census tract, census block group and exact place of residence units, Connecticut, 1988–92.

Significant, but slight, global clustering of late stage breast cancer was found for analyses at the block group and tract level, but no clustering was found when analyzed according to town. According to Figure 4, the spatial scan statistic was consistent in not locating statistically significant clusters with high proportions of late stage disease when cases were analyzed according to exact place, census tract or town of residence. However, analysis by block group found one area of Central Connecticut where late stage cases were 1.61 more likely among diagnosed cases than elsewhere around the State (p < 0.05).

## Discussion

Spatial analysis of health necessarily addresses issues about the accuracy of geocoded data, the requirements of time and training necessary to complete tasks, the threats to protecting confidentiality of sensitive health records and the interpretability of results for given areal units of analysis. Desire for greater precision challenges data safeguards as well as the technical capacity of available GIS systems. Surveillance by aggregating records into large areal units will yield greater proportions of accurate and protected records but possibly at the expense of capacity to identify discrete locales with elevated rates/proportions of health outcomes [16].

Our effort to contrast geographic analyses of prostate and breast cancers according to differing aggregation units across Connecticut yielded much, but not complete, consistency across analyses. Like others [20,22], we found in most instances that results obtained by block group level data mirrored those based on the census tract. As such, interpretations based on geocoded data available through the CTR were not appreciably enhanced by our further efforts to specify finer levels of geography. Global clustering of incident prostate and breast cancer cases was apparent for either level of geography and the test for local clustering found approximately the same locales, populations at risk and estimated effects.

On the other hand, some discrepancies were uncovered. Secondary cluster locations varied by level of analysis. More importantly, analysis of breast cancer incidence by town yielded an approximate location of a significant primary cluster some distance from results based on block group or tract. It is possible that discrepancy is not a product of analytic scale but the consequence of differing ability to geocode records across all locales [25]. Test of this hunch requires analyses whereby cases excluded from one level of analysis would be excluded from all other analyses. As our intention was not a pure test of MAUP but a 'simulation' of the choice investigators might confront when selecting between a geographically referenced files in hand (CTR generated) or one independently created using original address data, we did not pursue this line of inquiry here.

The local tests for late stage prostate cancer produced similar findings of significant clustering for analysis by exact coordinates, block group, tract or town, whereas results for the global clustering test were not significant for all but the block group analysis. Significant global clustering of late stage breast cancer was found using block group or tract, but not town or exact coordinates; significant local clustering was found only for the block group level analysis. Divergence across analyses could reflect distinctions among the levels of aggregation or merely subtly differences in the relative size of our data sets. It is noted that analysis of disaggregate (point) data raise issues separate from those specific to MAUP which we specifically address in this paper. It goes without saying that statistical procedures predicated on disaggregate (point) data would be unavailable if only aggregate files were available [26].

When analyzing geographic health data, concern regarding scale effects attributable to MAUP is unavoidable. Increased aggregation of data reduces power to detect very small clusters but stabilizes rate estimates. For now, the magnitude and direction of artifact generated by a given areal unit cannot be reliably predicted. Consequently, analysts will continue to be driven to select a preferred areal unit for analysis based on pragmatic rather than scientific consideration. In the absence of conditions calling for surveillance of small area cancer clusters ('hot spots'), the rationale for analysts to accept the technical, political and substantive burdens of preparing data at levels of geography finer than the census tract may not be compelling. The added protections to personal health data, the ease of interpretation and the applicability of similarly structured census and survey data organized argues for geographic studies to prioritize census tract level analyses.

## Methods

The geographies of breast and prostate cancer incidence in Connecticut, 1988–1992, were evaluated in relation to the State's populations-at-risk within towns, census tracts and block groups for 1990 (1,160,886 males, and 1,282,917 females 20+ years of age according to seven age-categories: 20–29 years, 30–39, 40–49, 50–59, 60–69, 70–79, 80+) [27]. The at-risk populations are predominantly white (89.1%) and concentrated along Connecticut's southern shoreline and central river valley; eastern and northwestern sections of the State are considerably less densely populated. As shown in Table 3, Connecticut's population is spatially organized within its 12,550 square mile area according to, 169 municipalities (towns), 834 census tracts and 2,905 block groups, as well as 50,569 census blocks, 330 zip codes, eight counties and two telephone area codes.

**Table 3:** Spatial and population characteristics of selected areal units of Connecticut.

Unit	Places	Area (sq. km)	1990 Population 20 & Over	Persons 20 + years per sq. mile
State	1	12,550	2,443,803	195
County	8	956 to 2,383	72,931 to 635,829	54 to 382
Town	169	13 to 160	443 to 100,552	6 to 2,426
Zip code	263	0.5 to 249	19 to 45,623	9 to 3,943
Census tract	834	<0.01 to 160	0 to 7,507	6 to 9,077
Block group	2,905	<0.01 to 86	0 to 5,415	0 to 21,333
Census block	50,569	Not available	0 to 2,796	Not available

Between 1988 and 1992, the Connecticut Tumor Registry (CTR) recorded incidence and stage of diagnosis of 10,054 invasive cancers of the prostate (ICD-9-185) and 12,518 breast cancers (ICD-9-174) among State residents. The Institutional Review Boards of the University of Connecticut and Connecticut State Department of Public Health approved our access to, and analysis of information reported here.

Geographic analyses of incidence by town and census tract were based on geographically referenced data files provided to us by the CTR. Every record identified an individual's town of residence and most assigned a census tract of residence to records (98% of prostate and 87% of breast cancer records). Total case counts are presented in Table 4. Why some records were not assigned census tract identifiers by the CTR could not be determined here.

**Table 4:** Geocoding of incident prostate and breast cancer cases, Connecticut, 1988–92.

	Prostate Cancers		Breast Cancers	
	Cases	%	Cases	%

Incident cases with town of residence recorded by the Connecticut Tumor Registry (CTR)	10,054	100	12,518	100
Census tract of residence recorded by CTR	9,825	98	10,924	87
Geocoded block group & street address of residence	9,207	92	11,864	95
Geocoded street address on 1^st ^try (stringent criteria)	4,546		5,926	
Geocoded street address on 2^nd ^try (relaxed criteria)	4,661		5,938	
Nursing home resident excluded for analysis by block group and exact coordinates	179		111	
Record not geocoded	847	8	654	5
Post Office box listed	178		64	
No street address listed	216		534	
No house number listed	176		23	
Listed address unable to geocode	277		33	

To examine if geographic patterns of cancer incidence and late stage change at finer units of analysis, we subsequently used the full street address available within the CTR record to independently assigned latitude-longitude coordinates to census block group and place of residence for 9,207 prostate (92%) and 11,864 breast (95%) cancer records. Our purpose was neither to augment nor correct the CTR data, but to generate separate geographically-referenced files to study cancer patterns according to aggregation units otherwise unavailable to external researchers. This accounts for the seemingly incongruous observation that 11,753 records were geocoded (by us) to block group whereas only 10,924 records were geocoded (by CTR) to tract. The result of our effort, vis-à-vis data provided by the CTR, is summarized in Table 4. As there is no 'gold standard' available to validate geocoded results, no effort was made to enumerate or resolve ambiguities that could be noted if files were directly compared.

Approximately one-half of records geocoded in this manner were categorized using stringent coding criteria (i.e., an address conforms completely to a street location recognized by geocoding software); the remainder were completed using 'relaxed' procedures (i.e., an address bearing one or more incongruities was assigned to the 'most likely' street location by the geocoding software) [28]. We were unable to geocode 847 prostate and 654 breast cancer records because only a Post Office box was available, no street or house number was recorded or the recorded address could not be matched to a recognized street location. Records for individuals with addresses associated with nursing home were not included in this phase of analysis (179 prostate and 111 breast cancer records, respectively); leaving totals of 9,028 prostate cancers and 11,753 breast cancers for study.

Numerous tests for spatial randomness (i.e., are geographical patterns due to random fluctuations/chance or true underlying variability?) are available [29]. For purposes of illustration, we selected one *global clustering *and one *cluster detection *test to evaluate geographic variations of disease rates.

Oden's Ipop [23] indicates whether there is an overall pattern of spatial aggregation of cases throughout the study region, without regard to specific locations where aggregation might occur. Group data are used to generate a weighted correlation coefficient, adjusted for population size, that indicates the extent to which case counts within given locations are associated with values of neighboring locales (i.e., are places with high frequencies adjacent to places with similarly high frequencies?). The significance of the computed value is evaluated in relation to an expectation derived by a hypothetical null spatial distribution of data. Oden's Ipop was calculated using *ClusterSeer v2.06 *software [30].

The spatial scan statistic [24] looks for significant concentration of cases at specific locations within a study region without preconceptions about where concentrations might be found. The spatial scan statistic utilizes scanning circles of varying location and size so as to contain 0–25% of the State's population at risk to identify places where the number of observed cases exceeds expectation under a null hypothesis that incidence is proportional to population density. The spatial scan statistic was calculated using *SaTScan 3.1 *[31].

Among the available address matched records, 9,207 (92%) prostate cancer and 11,864 (95%) breast cancer records contained sufficient information for geographic analyses of 'late stage' disease across the State. Historical SEER summary stage classifications [32] were used where regional/distant prostate or breast cancers were noted among 2,198 (28%) and 4,119 (40%) records, respectively. Analyses of geographic distribution of disease stage (regional/ distant versus local) using Oden's Ipop and the spatial scan statistic were completed according to town, census tract and census block group of residence. The spatial scan also was applied using exact place of residence coordinates of cases; because necessary group boundaries for discrete residential locations are unavailable, Oden's Ipop could not be used with individual coordinates. *Maptitude 4.5 *software [28] was used to map cluster locations with markedly high incidence rates (Figures 1 and 2) or proportions of late-stage disease (Figures 3 and 4).

## Authors' contributions

DG and MK conceived of the study; DG supervised all aspects of its implementation. LD and HS assisted with the study and completed the analyses. MK assisted with the study and analyses. DG, LD, and HS participated in drafting the manuscript. All authors helped review the manuscript and approved the final version.
